# *Glomhopper*—a subfamily of DUF3504-encoding *CryptonA* elements in Glomeromycota

**DOI:** 10.1186/s13100-026-00404-0

**Published:** 2026-06-06

**Authors:** Marianna Krysińska, Drishtee Barua, Anna Muszewska

**Affiliations:** 1 Institute of Biochemistry and Biophysics, Polish Academy of Sciences, Pawinskiego 5A, 02-106 Warsaw, Poland; 2 Warsaw University of Life Science - SGGW, Nowoursynowska 166, Warsaw, 02-787 Poland; 3 Doctoral School of Molecular Biology and Biological Chemistry at IBB PAS, Pawinskiego 5a, 02-106 Warsaw, Poland

**Keywords:** Tyrosine recombinase, Glomeromycota, Eukaryotic transposons, ZMYM3-like, DUF3504 domain

## Abstract

**Background:**

Transposable elements drive genomic changes and are mobilized by specific nucleases. Among them are tyrosine recombinases (YRs), which mediate DNA cleavage and rejoining. YR-encoding elements, such as *DIRS*, *Ngaro*, *Crypton*, and *Starships*, occur in diverse eukaryotes and display characteristic terminal repeat structures that enable their mobility. Their activity in fungi results in large-scale chromosomal rearrangements, horizontal gene transfer, and the movement of genes for pathogenicity, symbiosis, and secondary metabolism. Other YR-elements underwent domestication giving rise to ZMYM transcriptional regulators in animals.

**Results:**

We identify and characterize the fungal members of the CryptonA lineage of tyrosine recombinase-encoding transposons, which we name *Glomhoppers*. These elements encode a DUF3504 domain that retains the conserved catalytic residues characteristic of active YRs. In contrast, many domesticated animal DUF3504 homologs lack key catalytic residues, whereas active CryptonA transposon-derived DUF3504 elements have also been reported in animals. Structural modeling suggested the presence of a putative DNA-binding groove, and phylogenetic analyses placed *Glomhoppers* as a well-supported subclade within the CryptonA lineage, together with domesticated ZMYM-like derivatives.

Across 72 Glomeromycota genomes, ~ 1,800 *Glomhopper* copies were identified, representing a subset of DUF3504-containing loci, mostly truncated or intronized, but ~ 25% lacked introns and maintained intact catalytic motifs, consistent with potential mobility. Genomic context analysis revealed their frequent localization within highly repetitive compartments, often alongside other transposon families. Expression data indicated that intronless variants respond to stress, reaching several-fold higher expression levels than intron-containing forms, especially in Gigaspora species. This is consistent with the possibility that a subset of *Glomhoppers* remains transcriptionally active and potentially mobilizable, although direct evidence of transposition is lacking.

**Conclusion:**

Our findings establish *Glomhoppers* as a novel subfamily of DUF3504-encoding CryptonAs. The lineage-specific distribution, intron variation, and stress-responsive expression of *Glomhoppers* suggest divergent evolutionary trajectories, potentially including both mobility and domestication. These elements expand the known diversity of YR transposons and highlight DUF3504 as a candidate domain for further functional and evolutionary studies.

**Supplementary Information:**

The online version contains supplementary material available at 10.1186/s13100-026-00404-0.

## Background

Transposable elements (TEs) shape the genomic landscape and are one of the drivers of speciation and diversity. TEs are referred to as either class I elements (retrotransposons) with a reverse transcriptase moving by a “copy and paste” mechanism or as class II elements, which move by a “cut and paste” mechanism (transposons) [28, 82]. Most classes of eukaryotic TEs are mobilized by an RNAse H-like nuclease with a DDE active site [43]. The second most common type of integrase is tyrosine recombinase (YR) with a CAT fold [70]. YRs share a DNA breaking-rejoining enzyme fold with topoisomerases. DUF3504-containing YRs, including *CryptonA*-like elements, are grouped into DNA-mend clan (CL0382) in the Pfam database (Mistry et al. [46]). YRs are present both in class I eukaryotic mobile elements such as *DIRS*, *Ngaro*, *VIPER* and in class II elements such as *Crypton* and *Starship* and on top of that they are typical nucleases in bacteriophages [17, 62–64]. The best known representative of the YRs is the *Cre* bacteriophage P1 integrase. DNA cleavage by *Cre* involves the nucleophilic activity of the tyrosine residue hence the name of the enzyme. The broad tyrosine recombinase active site comprises seven amino acids. Crucial for activity are tyrosine and histidine [15]. The hydroxyl group of tyrosine attacks the phosphodiester bond of DNA, leading to the cleavage of one DNA strand. A covalent bond is formed between the tyrosine and the 3' end of the DNA and a phosphotyrosine intermediate is formed. The hydroxyl groups of the 5′-released by the cleavage of the phosphodiester bonds become nucleophiles in the strand transfer reaction, in which they attack the phosphotyrosines of the partner substrates to give the Holliday intermediate compound. A second round of cleavage and strand exchange reactions yields recombination products. During this time, histidine acts as a proton acceptor and stabilises the nucleophilic reaction of tyrosine. The two arginines and lysine form hydrogen bonds with the phosphate groups of the DNA, stabilising the reaction and preventing DNA strand separation during rearrangement. Glutamate/aspartate interacts with the nitrogenous bases in the inverted loxP repeats—during the rearrangement of strands, it stabilises the DNA conformation and ensures its correct orientation [15, 20, 24]. There are also known examples of modifications within this active site. For instance, in a related Cre/loxP recombinase Dre/rox, there are differences in three of the key amino acids: lysine is substituted by arginine, one of the arginines is substituted by proline and lysine is observed at the glutamate site [71]. Mobile elements with a YR depend on terminal repeats for their mobilization [19]. These elements display diverse types of terminal repeats. *DIRS* and *Ngaro* elements possess split direct repeats (SDRs) rather than classical LTRs, with many DIRS families exhibiting similar SDR structures [64]. *Cryptons* are not associated with terminal inverted repeats but instead are flanked by short direct repeats (DRs), typically 4–6 bp in length, generated upon insertion [63]. In contrast, some *Starship* elements possess terminal inverted repeats (TIRs), although these are longer and often asymmetric [17]. *VIPER* elements contain split direct repeats organized as A1 at the 5′ end and B1, A2 and B2 at the 3′ end [64]. Some *Crypton* families lack a repeat description.

Fungal genomes have a variable share of TEs ranging from several copies in yeast-like fungi to over half of the whole genome in rusts. In general, the abundance of TEs is positively correlated with genome size [47, 51]. The collection of fungal mobile elements is dominated by Ty3 LTR retrotransposons, *LINE-like* non-LTR retrotransposons and *Tc1/Mariner* transposon superfamilies [6, 48, 53]. Other class II transposons, such as *hAT, PiggyBac, PIF-Harbinger* and *MULE* most probably constitute a large, but underestimated, part of the fungal genomic sequences [50]. Several plant pathogens and plant related symbionts such as Glomeromycota, have transposon-rich compartments in their genomes [47, 57]. These regions are usually associated with accessory regions of fungal chromosomes, although the majority of TE insertions are relatively young, and their distribution may reflect ongoing dynamics rather than ancestral patterns [42].

Fungal genomes harbour several classes of TEs, which are equipped with a YR such as *Cryptons* [19], *DIRS* and *Ngaro* [49] as well as giant *Starship* elements [17]. *DIRS* and *Ngaro* resemble classical LTR retrotransposons in their overall organization and encode a reverse transcriptase. However, instead of typical long terminal repeats they contain non-canonical terminal structures. *DIRS*-like elements possess inverted terminal repeats (ITRs) and an internal complementary region (ICR), and in addition often encode a conserved methyltransferase (MT) domain downstream of the RT/RH. In contrast, *Ngaro*-like elements are characterized by split direct repeats (SDRs), composed of A1 at the 5′ end and B1, A2 and B2 at the 3′ end, and they lack the MT domain found in *DIRS*-like elements [13, 63]. They occur mostly in Mucoromycota and Basidiomycota genomes with *Ngaro* present preferentially in rusts and *DIRS* in Mucorales representatives [49]. *Cryptons* are simple transposable elements with a YR and facultatively a second protein-coding gene with a DDE transposase (has only been reported in MarCry), C48 peptidase and HTH DNA binding domain. These elements are associated with short direct repeats, which appear to reflect microhomology with the target sites rather than genuine target site duplication. A tentative model of mobility was proposed by Goodwin and co-workers [19]. *Cryptons* were identified in diverse fungal lineages including filamentous Ascomycota [62]. *Starships* are massive elements holding a set of cargo genes and leading to the formation of new direct repeats (DRs, which were originally annotated as target site duplications, TSDs) [17, 75]. *Starships* seem to be unique to filamentous Dikarya, they were discovered in Pezizomycotina and this subphylum is likely where they originated [17, 74, 80] Gluck-Thaler and [18]. Their YRs is referred to as *captain* (DUF3435), and this gene is often followed by a set of cargo genes. These genes contribute to fungal adaptability which was documented among others in *Aspergillus fumigatus* [16], *Paecilomyces* [75] and *Penicillium* strains [55]. The role of cargo-less elements is more subtle, however even non-coding MITEs have been linked to pathogenicity in *Fusarium* and are localized in the promoter regions of effector genes [67]. This highlights the role of TEs not only as passive genomic passengers but as active drivers of evolutionary innovation, creating lineage-specific adaptations [41].

In this study, we identify and characterize a novel group of tyrosine recombinase transposons, which we name *Glomhoppers*, because they are present mainly in Glomeromycota and their closest relatives. *Glomhoppers* represent a distinct subgroup within the *CryptonA* lineage of YR-utilizing mobile elements. *Glomhoppers* belong to the broad diversity of YR-utilizing mobile DNA elements that also includes *Cryptons*. We use the term Crypton here in its original sense, i.e., as a eukaryotic DNA transposon encoding a tyrosine recombinase, without implying a single common origin for all *Crypton* families. Importantly, *Cryptons* do not represent a single monophyletic lineage but comprise several distinct superfamilies, often characterized by different accessory Pfam domains, and are united mainly by the use of YR-mediated recombination for mobilization [31, 32]. Unlike classical DNA transposons, *Cryptons* are generally not associated with target site duplications (TSDs); instead, they are flanked by short direct repeats complementary to their insertion sites, reflecting a site-specific recombination mechanism originally proposed by Goodwin [19]. Although this structural organization has been interpreted inconsistently in the literature, recent genomic studies have provided strong evidence that both Starship elements [75] and *CryptonF* elements [40] operate according to this model.

Some animal DUF3504 proteins, particularly in tetrapods, have been suggested to represent domesticated derivatives of *Crypton* elements [32]. However, our phylogenetic analyses indicate that *Glomhoppers* form a well-supported subclade within the *CryptonA* lineage, rather than a lineage separate from previously described *Crypton* groups. *Glomhoppers* exhibit dynamic variation in copy number across Glomeromycota isolates and widespread intronization, suggesting both ongoing mobility and possible domestication processes. These findings extend the diversity of known eukaryotic YR transposons and provide a new evolutionary context for DUF3504-containing elements.

## Results

### Characterization of the DUF3504 tyrosine recombinase in fungi

*Fungi also have DUF3504 homologs*. The domain of unknown function DUF3504 (PF12012) is distantly related to other tyrosine recombinases (YRs). Proteins with a DUF3504 domain are widespread in Opisthokonta; most animal genomes from Mollusca and Porifera to mammals have several DUF3504 encoding genes. Fungal copies consistently retain the conserved catalytic residues shared with known and crystallized YR integrases such as *Cre* [20], However, several non-fungal homologs with intact YR catalytic motifs have also been reported [32], including examples from medaka, sea urchin, acorn worm and sea anemone. At the same time, many DUF3504 domains in animal proteins lack the conserved residues typical for tyrosine recombinases and were therefore previously proposed to function as transcriptional regulatory domains [32, 60].

Most fungal DUF3504 (PF12012) homologs have been detected in a single fungal lineage—the Glomeromycota phylum. Members of the Glomeromycota form arbuscular mycorrhizas (AMs) with the thalli of bryophytes and the roots of vascular land plants [9, 58]. In consequence, we define a new subfamily of YR transposons with a DUF3504, referred hereafter as *Glomhoppers*. *Glomhoppers* represent transposon-encoded DUF3504 proteins and do not include host-encoded or otherwise domesticated/non-functionalized copies. *Glomhoppers* can be found in all of the sequenced genomes of the representatives of Glomeromycota, both in Diversisporales and Glomerales. In addition, a small number of *Glomhopper*-like elements were also detected in the genomes of representatives of Mucoromycota in our analysis, including *Fennellomyces* spp. and *Phascolomyces articulosus*.

**Fig. 1:**
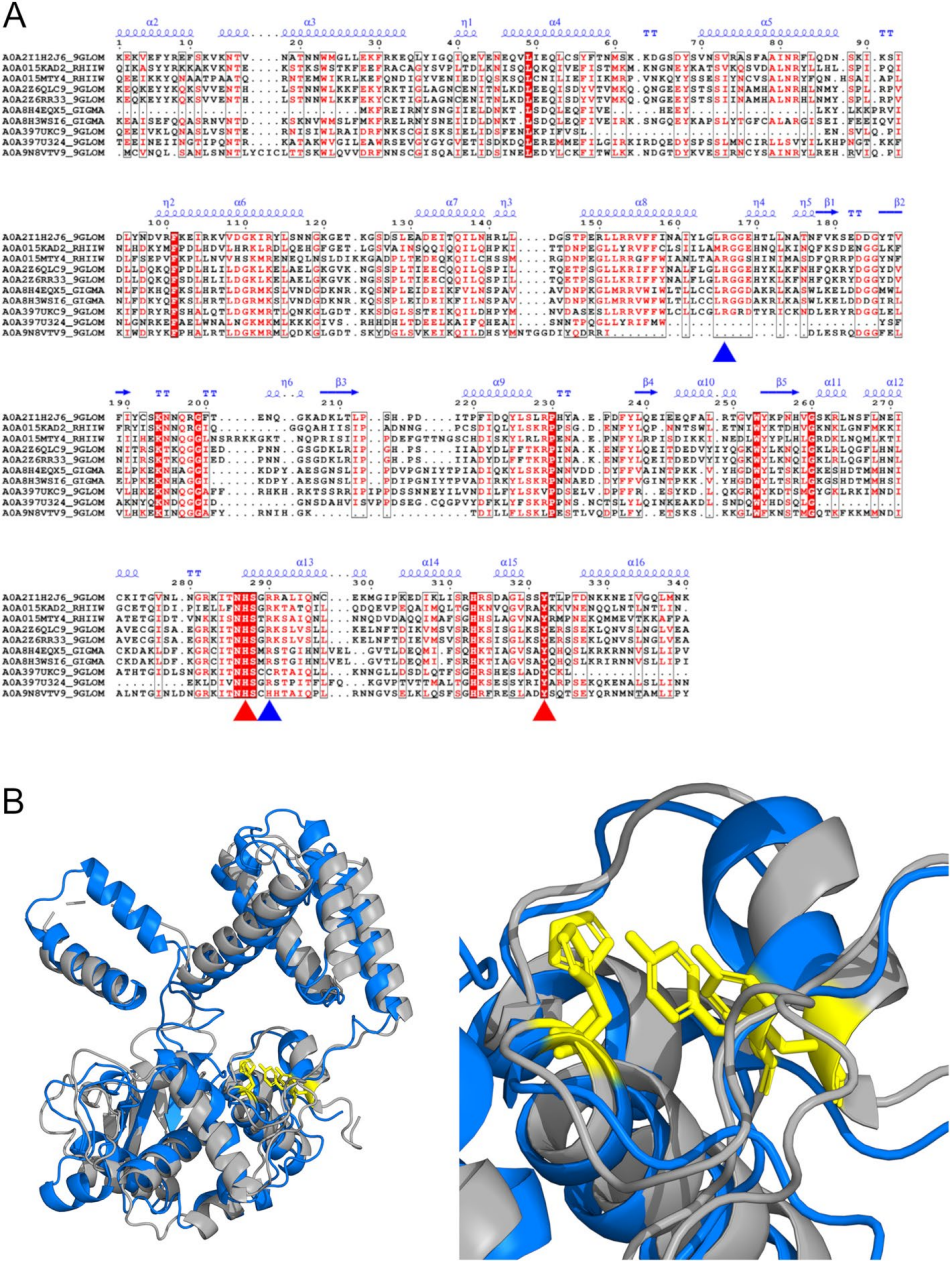
Conservation of sequence and structure features of recombinase in representatives of the *Glomhopper* family. **A** Alignment of 10 representative DUF3504 sequences from *Rhizophagus irregularis* (strain DAOM 197198w), *Rhizophagus clarus*, and *Gigaspora margarita*, *Gigaspora rosea* and *Funneliformis caledonium*. The CRE recombinase is included as a structural reference and shown in the alignment in the form of secondary structure annotation rather than as a full-length aligned sequence. Legend: red triangles denote critical important residue and blue triangles mark supporting residues; red box with white character means strict identity; red character means similarity in a group; black frame means similarity across groups. **B** Alignment of protein structures: AlphaFold3 model of DUF3504 domain from *Rhizophagus*—A0A2I1H2J6 (blue) and CRE recombinase 7RHX (grey). Conserved catalytic residues (histidine and tyrosine) are shown in yellow. Alignment of protein structures—FatCat server [39]

*DUF3504 is predicted to be an active tyrosine recombinase*. AlphaFold [77] models of fungal DUF3504 proteins suggest a conserved protein structure and a putative DNA-binding pocket/groove near catalytic residues (see Fig. 1). The active site of the known catalytic tyrosine recombinase domain consists of seven, usually conserved, residues: two arginine and histidine residues, aspartate or glutamate, lysine and an essential catalytic tyrosine residue. In particular, Tyr and Lys serve as nucleophilic and general acid catalysts, respectively. Two Arg residues neutralise the negative charge in the transition state and activate the scissor phosphate via the catalytic tyrosine residue. Two His and Glu/Asp residues stabilize the transition state. Among these, the R-H-R-Y tetrad is considered the hallmark of the recombinase family [54]. Structural alignment of DUF3504 and known YR integrases confirm the conserved positions of catalytically key histidine and tyrosine (in CRE H289 and Y324) [15]. In agreement with previous analyses [32] many DUF3504-containing proteins from animals lack a conserved catalytic region; in numerous cases neither the key histidine nor the catalytic tyrosine are preserved, consistent with likely loss of recombinase activity. In contrast, fungal DUF3504 proteins consistently retain these residues and are therefore predicted to be catalytically active.

### *Glomhopper* classification based on YR sequence

To observe the relationship between DUF3504 and other YR sequences we conducted clustering of representative DUF3504 homologs together with known YR retroelements. Through the use of the CLANS algorithm, we have established *Glomhopper's* links to the DNA_mend clan described in the Pfam database. *Glomhopper* shows distant similarity to the previously described *Crypton* transposon, as well as to prokaryotic YRs.

**Fig. 2:**
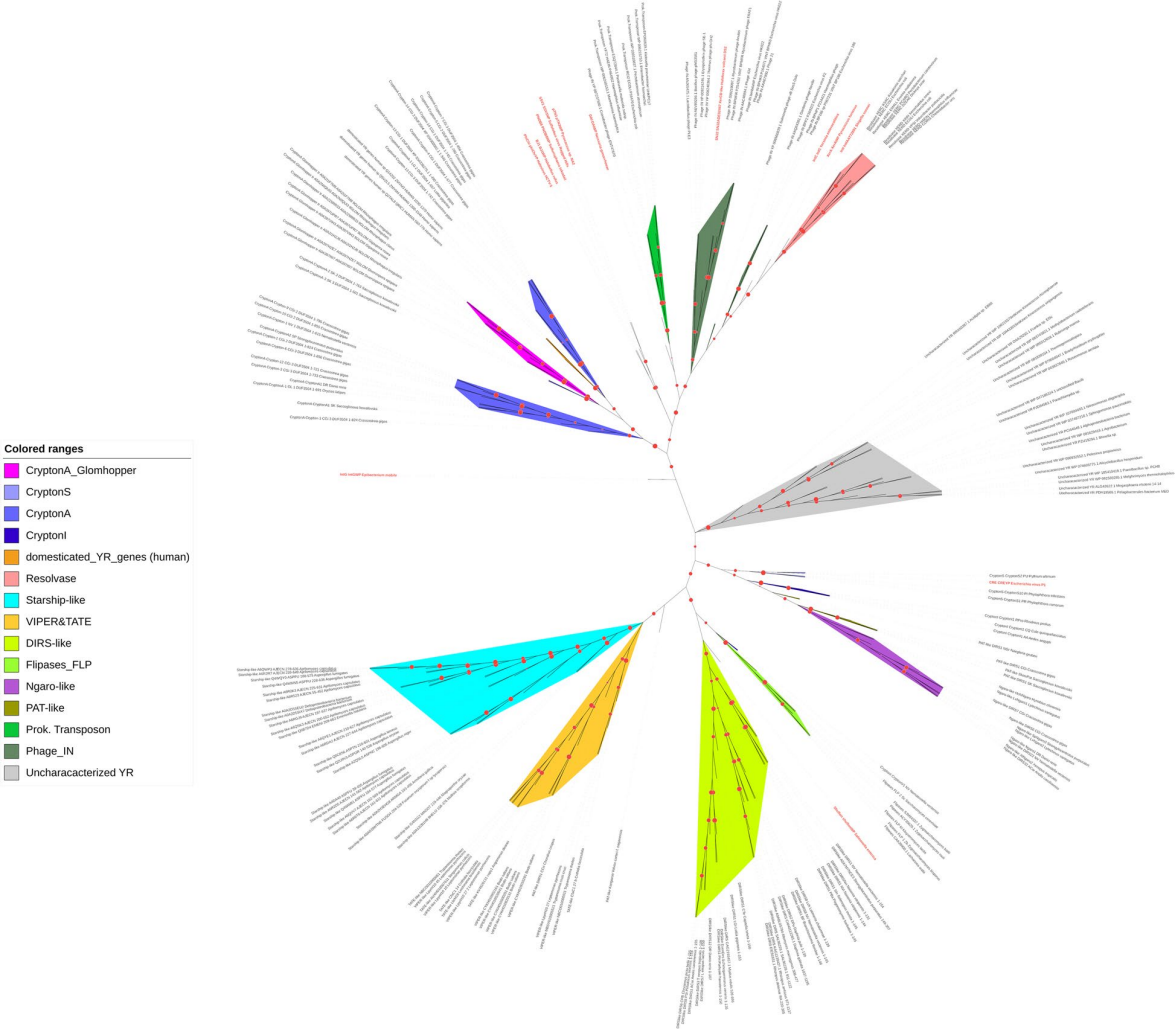
Phylogenetic relationships of YR family proteins. Unrooted ML tree (VT + R5) based on the YR amino acid sequences from *Glomhopper* transposon-encoded DUF3504 proteins along with other YR family proteins described in [64] in suppl 18: resolvase [site-specific recombinases involved in chromosome dimer resolution in archaea (XerA) and bacteria (XerC/D)], FLP (yeast Flp-like flippases), phage integrase, prokaryote transposons, *Crypton*, uncharacterized YR (proteins retrieve by blastp searches) *VIPER*&*TATE* and, in addition, *Starship*. YR sequences from the distinct groups shown by Wang et al. (2018) are in red: shufflon (shufflon-specific DNA recombinases); DAI (dusA-associated integrases); phage integrases; IntC (integrases from integrative and conjugative elements); IntI (integron integrases); IntG (genomic island integrases); XerA (site-specific recombinases involved in chromosome dimer resolution in archaea), Cre (phage P1-like recombinases); archaeal SSV-type, pNOB8-type and pTN3-type integrases; and integrases encoded by haloarchaeal SNJ2, BJ1-like and phiCh1-like viruses. Results of bootstrap statistics from ML analysis are presented like red dots on a branch (values ≥ 50 are shown; only strongly supported nodes are discussed in the text)

**Fig. 3:**
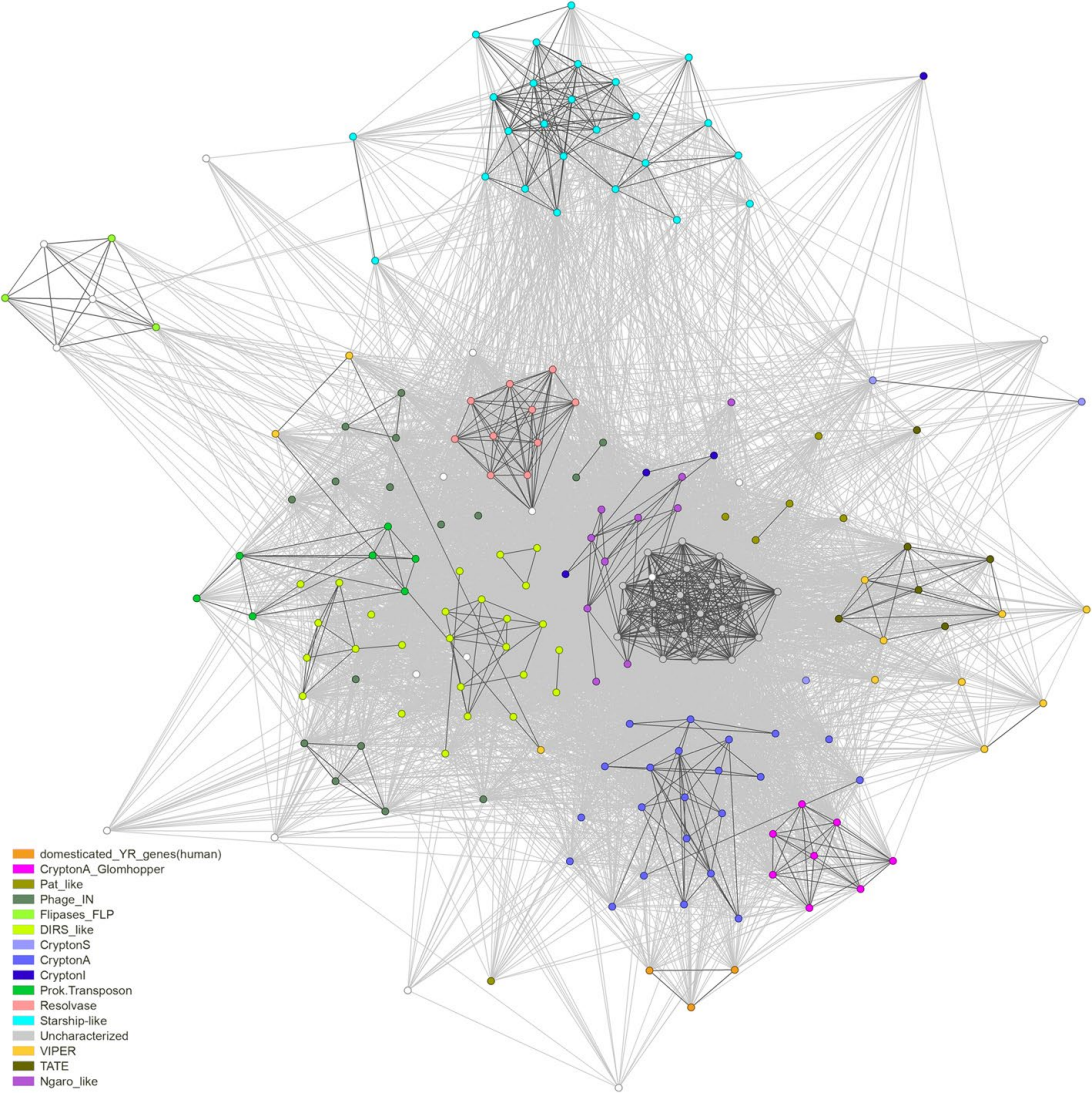
CLANS graph of selected DNA_mend Pfam set of YR. This CLANS graph was generated using the same dataset as the phylogenetic tree presented earlier. It includes representative sequences from the DNA_mend Pfam family of tyrosine recombinases (YR), with *Glomhopper* elements highlighted in turquoise, human DUF3504-containing proteins in brown, and *Starship* YR–DUF3435 in pink. The graph illustrates sequence similarity-based clustering, providing a complementary view to the phylogenetic analysis

Phylogenetic analyses indicate that *Glomhoppers* form a distinct fungal clade within the broader *CryptonA* lineage, previously described mainly from animals. To provide a consistent classification of *Glomhoppers* and to gain more insight into the evolution of YR retroelements, we performed phylogenetic analyses of representative elements. *Glomhopper* elements form a well-supported subclade within the *CryptonA* group and are clearly separated from neighbouring lineages, including the domesticated *Crypton*-derived ZMYM3-like DNA-binding proteins. *Glomhopper* and human DUF3504 homologs are well separated on the resulting unrooted phylogenetic tree (Fig. 2) and clustering image (Fig. 3), forming two distinct subclades with bootstrap support values indicated on the nodes (≥ 50). Mammalian DUF3504 homologs have previously been recognized as domesticated *Crypton*-derived integrases [32], but according to our phylogenetic analysis, as well as previous published YR phylogenetic trees [64], they appear more closely related to *Viper* and phage integrases than to other *CryptonA*-associated sequences. Consistent with previous studies [32], these domesticated mammalian homologs typically lack the conserved R-H-R-Y tetrad and are likely inactive. In contrast, transposon-derived DUF3504 elements identified in animals retain catalytic residues in their consensus sequences. Fungal *Glomhoppers* retain these residues and are predicted to be catalytically active. Active (fungal) and inactive (mammalian) DUF3504 form a separate group within the superfamily.

**Fig. 4:**
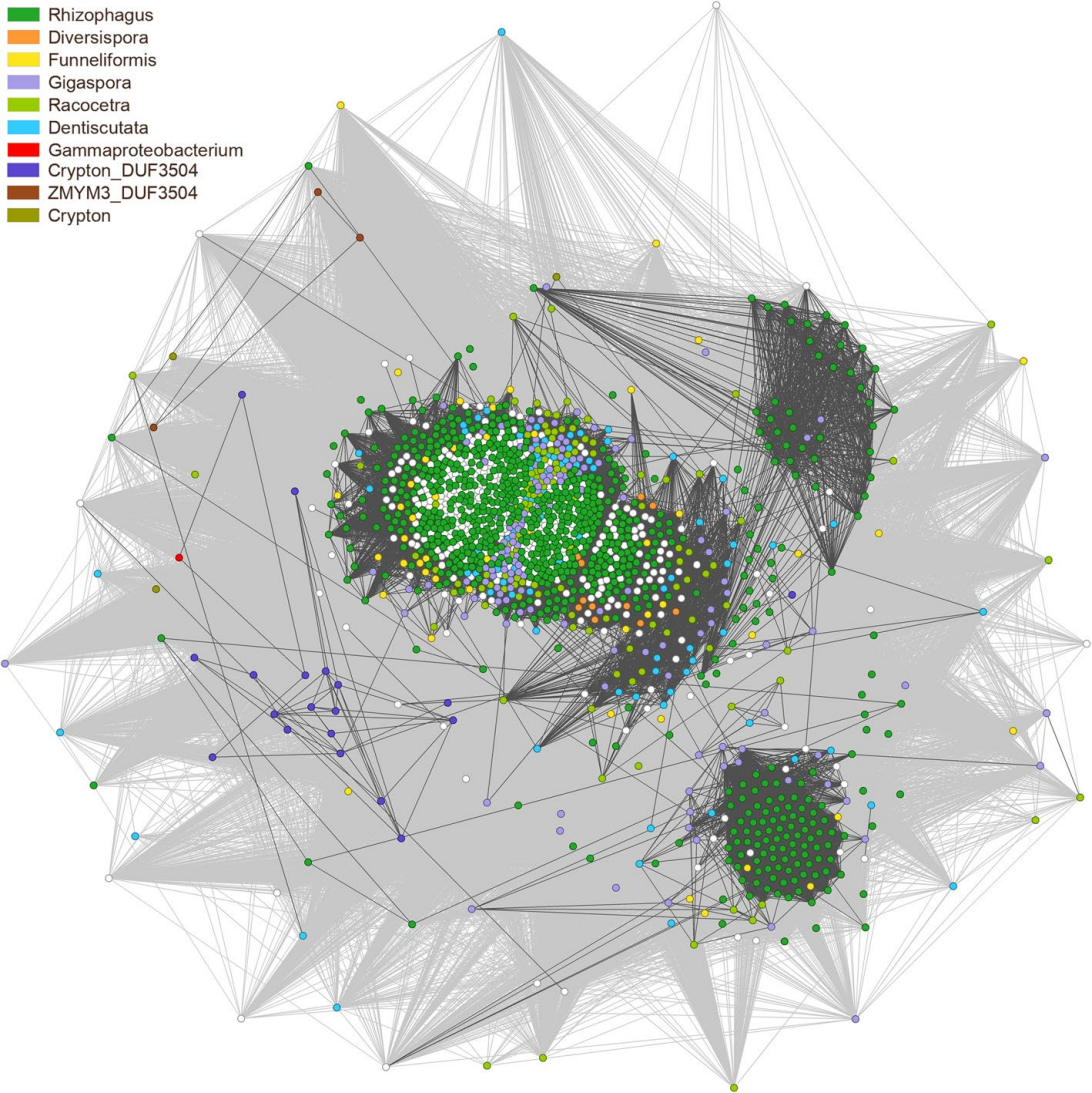
CLANS graph analysis of DUF3504 homologs. The analysis was performed on 1,594 representative amino acid sequences obtained from *Mucoromycota* genomes. Sequences were initially identified by RPS-tBLASTn using the DUF3504 domain profile from the CDD database, resulting in 9,609 hits. These sequences were then clustered with CD-HIT at 95% identity, yielding 8,997 non-redundant sequences. For the CLANS analysis, only sequences longer than 50 amino acids and with an E-value ≤ 0.005 were retained. CLANS was run with an E-value threshold of 1e-2. Sequences were coloured by taxonomy with RepBase references and animal ZMYM3-like proteins.The 1,594 sequences used in the CLANS analysis represent a nonredundant subset of all DUF3504 hits reported in Materials and Methods

Some sequences previously annotated as *Cryptons* are not monophyletic and form several separated groups both in sequence clustering (Fig. 3) and in phylogenetic tree (Fig. 2), which is consistent with earlier reports on the possible multiple acquisition of bacterial transposons [31]. *CryptonA* encompasses *Cryptons* with a DUF3504, including a potentially active subfamily of *Glomhoppers* which form a well-supported fungal clade Fig. 4).

The Glomhopper sequences analysed exhibit moderate sequence diversity (~ 24%), yet they form a well-supported monophyletic clade within the broader *CryptonA* lineage. Within this clade, clustering analyses and phylogenetic reconstruction reveal several distinguishable subgroups, which may be considered putative “subfamilies” based on sequence similarity. Most subgroups are conserved across multiple Glomeromycota species, while a smaller fraction appears to be lineage- or species-specific, reflecting recent diversification or expansion events. Examination of the DUF3504 domain indicates that the majority of fungal *Glomhoppers* retain the catalytically essential residues, consistent with a potentially active tyrosine recombinase. In contrast, mammal DUF3504 homologs lack these key residues, confirming previous reports of their inactive status. Cross-species comparison shows that *Glomhoppers* are widely distributed within Glomeromycota, with variation in copy number and subgroup representation among species, indicating both conserved and lineage-specific expansions. Overall, these results highlight the sequence diversity, subgroup structure, and partial conservation of the YR domain, providing a framework for future functional and evolutionary studies of *Glomhoppers* across fungal hosts.

### *Glomhopper* properties

The identified elements appear to be taxonomic lineage dependent in terms of copy number and length. Most YR copies were identified in *Rhizophagus irregularis*. They are often encoded by displaced or short contigs (58%) without identifiable repeats. *Glomhoppers* are clearly localised in repetitive parts of fungal genomes with Pkinase repeats, Sel1 and a multitude of other repetitive elements. Their genomic neighbourhood are regions of multiple 'nested' insertions of genomic fragments abundant with known transposons of different classes (*hATs*, LTR retrotransposons, *DDEs*, *MuLEs* and others), making it difficult to unambiguously identify the boundaries of *Glomhopper* and their possible repeats (see supplement).

While scanning 72 complete WGS of 34 Glomeromycota representatives stored in the NCBI assembly database, we have identified about 1,800 copies of *Glomhopper* transposons, most of which are either short, truncated or incomplete but still contain at least a fragment of the DUF3504 domain. To verify the structural boundaries of these elements, all assemblies were mapped to the reference genome of *Rhizophagus irregularis* (GCA_026210795.1; ASM2621079v1) using minimap2, which showed no detectable repeats assess the conservation of neighbouring sequences across lineages (Supplement S1, Table 6).

To further evaluate sequence redundancy and approximate the number of non-redundant genomic loci represented within these copies, we performed nucleotide-level clustering using CD-HIT-EST at different identity thresholds. At 95% identity, clustering resulted in 1,107 clusters, while 996 and 781 clusters were obtained at 90% and 80% identity thresholds, respectively (out of 4,402 total copies). This indicates that the observed copy number reflects a mixture of closely related but non-identical insertions rather than a set of nearly identical duplicated sequences.

### Expression and presence of introns

**Fig. 5:**
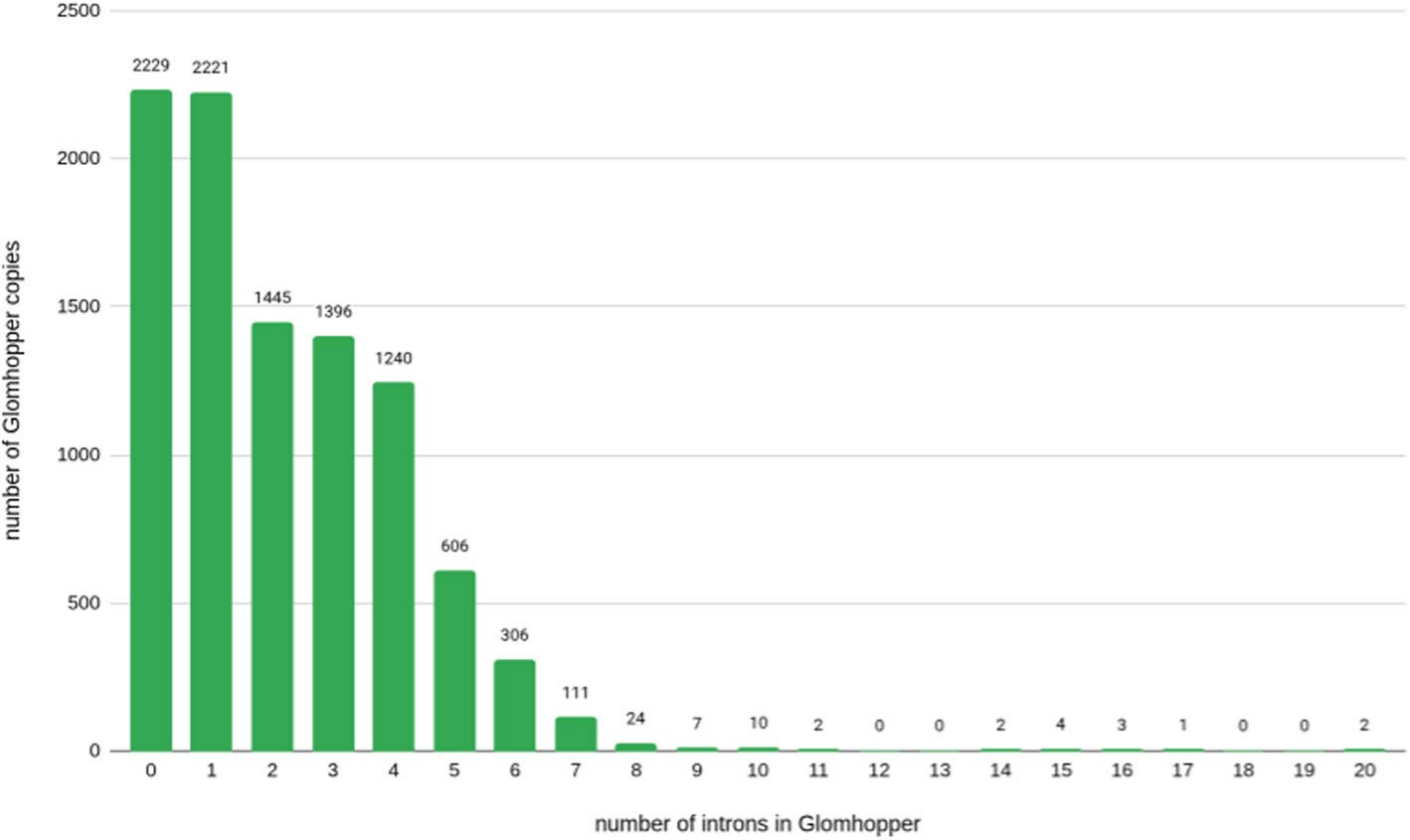
Histogram of intron abundance in 9,609 *Glomhopper* copies. The number of introns was predicted for 9,609 DUF3504 coding genes using METAEUK. This histogram includes all introns predicted by METAEUK, regardless of transcriptomic support. Only a subset of these introns is supported by RNA-seq data (see main text). No consistent intron positions were observed across multiple *Glomhopper* copies. (Supplement S1, Table 5)

In our set of *Glomhopper* sequences, 25% of candidate *Glomhopper* lacked introns and the remaining 75% had a variable number of introns ranging from 1 up to 20. (see Fig. 5 for a detailed distribution). Most elements had 1–3 introns. Predicted intron acquisition by transposable element–derived genes has previously been associated with functional diversification or domestication; however, the presence of introns alone does not necessarily imply loss of transpositional competence. Actively transposing elements often maintain compact, uninterrupted open reading frames, but DNA transposons can in principle tolerate introns provided that correct splicing occurs and an intact recombinase is produced. Thus, extensive intronization may reduce the likelihood of autonomous activity by affecting transcriptional efficiency or expression fidelity, but this effect remains inferential rather than demonstrative.

The histogram in Fig. 5 shows all introns predicted by METAEUK in 9,609 *Glomhopper* copies, regardless of transcriptomic support. Not all predicted introns were supported by transcriptomic data, as expression evidence was not detected for all loci. For downstream analyses, only introns supported by spliced RNA-seq reads spanning exon–exon junctions were retained to minimize annotation artefacts. Across the analysed transcriptomic datasets, the number of validated introns varied substantially between species (e.g. from 0 in *Ambispora leptoticha* to 141 in *Gigaspora rosea*), indicating that only a subset of predicted introns is supported by expression data. No intron positions were consistently shared across different *Glomhopper* copies, indicating that intron positions are largely variable. No evidence for aberrant or incorrectly spliced transcripts was detected in the analyzed datasets.

**Fig. 6:**
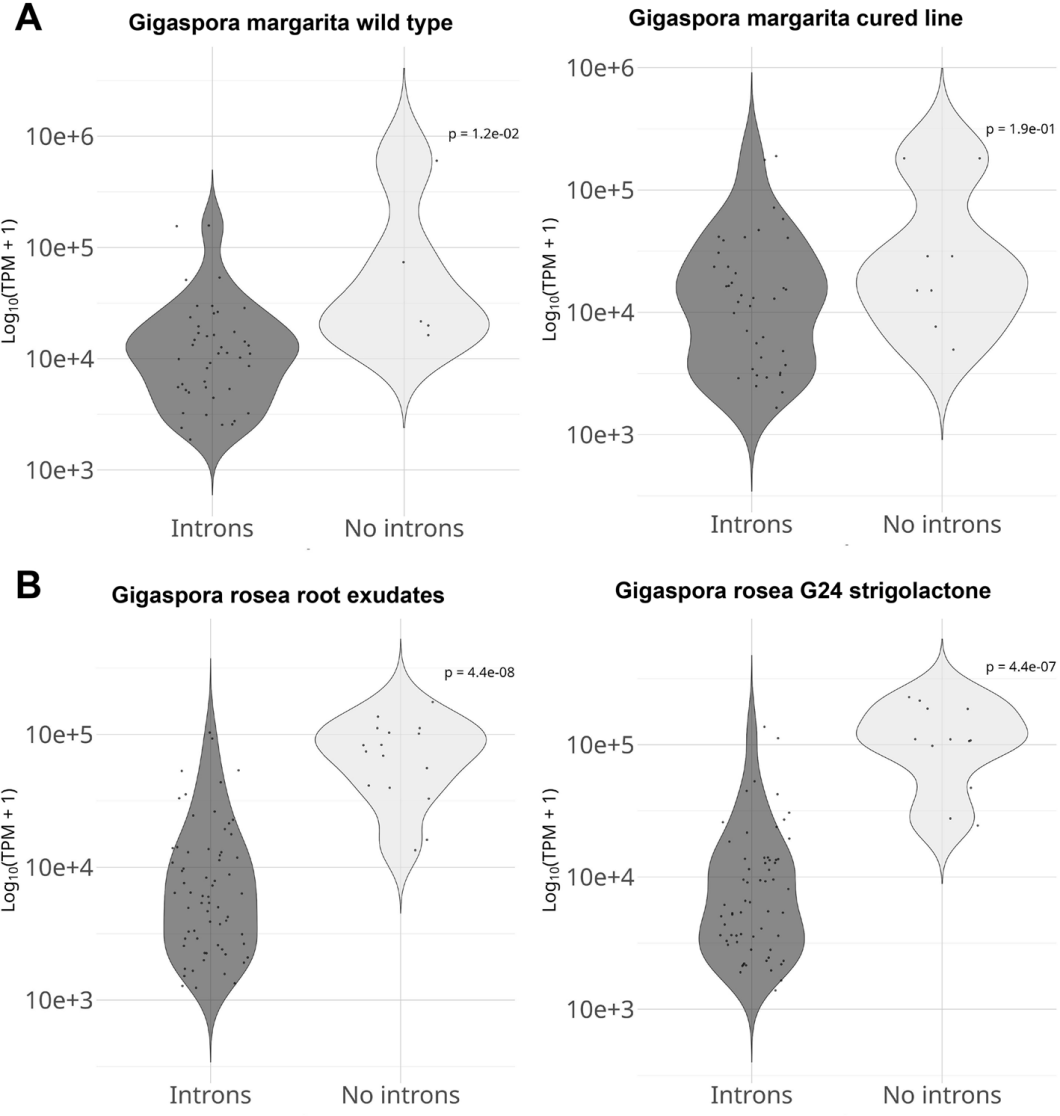
Differences in the levels of expression of *Glomhopper* copies with and without introns. **A** Out of the predicted 435 intron-containing and 111 intronless transcripts in *Gigaspora margarita*, 82 and 12 of them were validated using the transcriptomic dataset SRP049975. **B** Out of the predicted 314 intron-containing and 97 intronless transcripts in *Gigaspora rosea,* 141 and 27 of them were confirmed using the transcriptomic dataset SRP057297. The TPM values are shown in log10-scale. Each dot in the plot represents an individual *Glomhopper* transcript and its average TPM value. The width of the violins indicate the density of TPM values within each category. The expression levels between intron-containing and intronless transcripts were compared using the Wilcoxon rank-sum test to assess whether the differences in expression between the two groups are statistically significant. The p-value for statistical significance stands at *p* ≤ 0.05 [23]. **A**. *Gigaspora margarita* growth in association with *Candidatus Glomeribacter gigasporum* [66], and **B**. *Gigaspora rosea* response to plant signals in the switch from asymbiotic to presymbiotic growth (spores exposed to root exudates of *Daucus carota* roots and spores treated with GR24 synthetic strigolactone) [72]

Analysis of the expression levels of the two *Glomhopper* variants grouped by predicted intron presence revealed significant differences under several environmental conditions, most prominently in the genus *Gigaspora*. Variants lacking predicted introns during symbiotic growth reached 2–8 times higher expression levels than their intron-containing variants under the same conditions (Fig. 6A and 6B). *Gigaspora rosea* exposed to plant-derived signals showed significant differences in expression between intron-containing and intronless transcripts (Fig. 6B). In contrast, expression levels did not differ significantly between these two groups in the cured *Gigaspora margarita* line (p = 0.19), while a moderate difference was observed in the wild-type strain harboring Candidatus *Glomeribacter gigasporarum* (p = 0.012), suggesting that host-associated factors, including endosymbionts, may influence *Glomhopper* transcriptional regulation (Fig. 6B).

In the transcriptomes of *Rhizophagus* species, only intron-containing *Glomhopper* copies were detected, indicating that intronless copies may be strain-specific or located in genomic regions not represented in current assemblies (Table 1). Analysis of additional pure-culture transcriptomes from *Funneliformis mosseae, Acaulospora morrowiae*, and *Scutellospora calospora* (Table 2) showed that *Glomhoppers* are generally more highly expressed in symbiotic contexts, supporting transcriptional responsiveness rather than directly demonstrating ongoing mobility.

**Table 1:** Change in *Glomhopper* expression levels in condition-specific transcriptome data for different species in intron-containing and intron-less copies. The percentage changes in TPM values were calculated relative to the TPM of intron-containing transcripts

**Organism**	**Collected from**	**Type of study**	**Condition**	**Average of TPM values for intron-containing transcripts**	**Average of TPM values for intronless transcripts**	**Percent increase/decrease in TPM values; intronless v. intron**
Gigaspora margarita	[66]	Impact of endobacterial population in AMF on host transcriptome	Wild type	20,016	146,758	733%
Gigaspora margarita	[66]	Impact of endobacterial population in AMF on host transcriptome	Cured line	26,005	57,901	222%
Gigaspora rosea	[72]	Gene regulation in response to plant signals	Root exudate	12,950	77,988	602%
Gigaspora rosea	[72]	Gene regulation in response to plant signals	G24 strigolactone analog	13,916	120,848	868%
Rhizophag us clarus	NCBI Bioproject:PRJDB4082	Comparative transcriptome analysis of acid-tolerant and -sensitive AMF during polyphosphate accumulation	Intra radical mycelium	74,319	Not found	**-**
Rhizophagus irregularis	[26]	Transcriptomic profiles of R. irregularis extraradical mycelium (ERM) and intraradial mycelium (IRM)	Extra radical mycelium	174,318	Not found	**-**
Rhizophagus irregularis	[25]	Transcriptome profiling of extraradical tissues of R. irregularis	Germinated spores and hyphae	47,510	Not found	**-**

**Table 2:** Change in *Glomhopper* expression levels in pure transcriptome data for different species in intron-containing and intron-less copies. The percentage changes in TPM values were calculated relative to the TPM of intron-containing transcripts

Organism	Collected from	Average of TPM values for intron- containing transcripts	Average of TPM values for intron-less transcripts	Percent increase/decrease in TPM values; intronless v. intron
Scutellospora calospora	[4]	333,333	500,000	150%
Acaulospora morrowiae	[4]	1,000,000	1,000,000	100%
Funneliformis mosseae	[78]	500,000	333,333	67%

Our results suggest that the DUF3504 domain encoding regions occur in Mucoromycota in different structural forms, which may indicate evolutionary functional adaptations/neofunctionalizations. Rather than directly distinguishing “intact” versus “defective” transposons, the observed variation in predicted intron content likely reflects a continuum of evolutionary outcomes, including retention of transposon-derived functions, regulatory diversification, and potential domestication. The substantial fraction of intronless copies suggests that some Glomhoppers may retain the potential to encode uninterrupted recombinases, whereas the widespread presence of intron-rich variants is consistent with ongoing divergence and functional repurposing.

## Discussion

Characterized YR are classified into several Pfam families: Phage_integrase (PF00589) present in phages (e.g. *Cre*, P06956), *DIRS, Pat* and *Ngaro* retrotransposons, Archaeal phage integrase (PF16795), Integrase (PF12835), Integrase_2 (PF13009) and DUF3435 (PF11917) encoded by *Starship* giant transposons. There is also another, eukaryotic domain of unknown function, DUF3504, present in *Crypton* YR elements (Kojima and Jurka 32. Interestingly, the described *Crypton* YR copies possess neither TIRs nor conserved active site residues [32]. *CryptonA* YR encodes a single gene the YR (DUF3504) and was domesticated in animals to a ZMYM3-like DNA-binding protein family encoded by genes: *zmym2*, *zmym3*, *zmym4* and *qrich1* [32]. Among the domesticated YR-elements ZMYM3 is characterized and plays roles in chromatin remodelling and gene expression control [27, 32]. Such domesticated remnants illustrate how TE-encoded recombinases can be co-opted by the host genome to acquire new regulatory functions. While analogous cases in fungi remain less explored, the presence of diverse YR-encoding elements raises the possibility that similar processes of molecular domestication may have occurred, potentially shaping transcriptional networks and genome organisation.

DUF3504 encoding elements were previously identified by Kojima and Jurka [32]. Our results show that fungal DUF3504-containing elements exhibit variable or poorly conserved terminal repeats, consistent with the structural diversity previously reported for *Crypton* elements. Several lines of evidence nevertheless suggest recent or ongoing mobility of these elements in fungi. For instance, analysed fungal genomes differ in copy number among assemblies originating from related organisms, and more importantly genomic alignments show unique insertions missing from other assemblies. These observations indicate that fungal DUF3504-containing elements represent potentially active members of the *CryptonA* superfamily rather than fully domesticated derivatives.

We propose to designate the fungal DUF3504-containing *CryptonA* elements as *Glomhoppers*, reflecting their predominant taxonomic distribution in Glomeromycota and related fungi. The sequence similarity between the fungal *Glomhopper* homologs and animal ZMYM3-like DNA-binding proteins undoubtedly groups them into one Pfam family and disjointly from other YR recombinases such as Phage_integrase (PF00589) present in phages (*Cre*, P06956), *DIRS* and *Ngaro* retrotransposons and DUF3435 (PF11917) encoded by *Starship* giant transposons.

Phylogenetic analyses indicate that *Glomhoppers* form a sublineage within the *CryptonA* superfamily and fall within the diversity observed in animal *CryptonA* elements. Notably, the diversity of *Glomhoppers* is narrower than that observed within individual animal species such as *Crassostrea gigas* or *Saccoglossus kowalevskii*, suggesting that *Glomhoppers* likely originated in fungi via horizontal transfer from an animal *CryptonA* lineage.

Thus, fungal *Glomhoppers* represent a recently derived lineage of *CryptonA* elements that remain at least partially mobile in fungal genomes.

The structural variation of DUF3504 genes in analysed genomes shows divergent evolutionary pathways of *Glomhopper* copies post integration. Sequence analyses reveal that *Glomhoppers*, while forming a coherent fungal clade within *CryptonA*, exhibit moderate sequence diversity. Within this clade, several distinguishable subgroups or putative “subfamilies” can be defined based on sequence similarity, with some conserved across multiple Glomeromycota species and others appearing to be lineage- or species-specific. The majority of *Glomhoppers* retain the catalytically essential residues of the DUF3504 domain, indicating potential recombinase activity, whereas a smaller fraction shows disrupted or divergent sequences. Cross-species comparison also highlights variation in copy number and subgroup representation, reflecting both conserved and recently expanded elements. These patterns provide a framework to interpret *Glomhopper* sequence diversity, functional conservation, and potential evolutionary trajectories across fungal lineages.

Most of the DUF3504 genes are predicted to contain introns, and only one in four appears intronless. Although intronization and transcription of *Glomhopper* copies are consistent with post-integration diversification and may reflect early stages of functional co-option, our data do not allow us to unambiguously distinguish domesticated copies from degenerating, non-autonomous remnants of formerly active transposons. The lower expression levels observed for intron-containing copies are equally compatible with reduced transpositional competence, as active elements are expected to be more highly transcribed to ensure sufficient recombinase production. Thus, intron-rich *Glomhopper* variants may represent a heterogeneous pool including both transcriptionally permissive but functionally inactive copies and candidates for incipient domestication. Resolving these alternatives will require functional assays addressing recombinase activity, DNA cleavage capability, and potential host-specific roles of intron-containing DUF3504 genes.

The independent domestication and exonization of DUF3504 recombinase in both fungi and animals raises questions about the evolutionary pressure driving this phenomenon. Many TE exonizations reported so far have a restricted taxonomic scope, often occurring in specific lineages or subphyla rather than universally across all eukaryotes. For example, retroviral genes such as *env* in placental mammals [33], RNLTR12-int in the maturation of oligodendrocytes [14]) or DDE nucleases (recurrent domestications of piggyBac elements in animals [21]). By contrast, the domestication of ZMYM3/QRICH1 occurred early in the bilaterian ancestor, representing a much broader phylogenetic distribution. Importantly, known cases of exaptation include genes with a retained nuclease activity [3] and those which provide a structural scaffold for protein interactions (Bischerour et al. [5]) or DNA binding capabilities like ZMYM3-like genes. In the case of *Glomhopper* it is likely that the exapted copies retained the ability to cleave DNA because of the conserved residues present in the predicted active site of the encoded protein. The shape of the active site pocket and composition of key amino acid residues closely resemble those in *Cre* recombinase.

Importantly, Glomeromycota symbiotic lineages apparently lost many recombination and repair genes [69, 79]. It is an open question whether an exapted *Glomhopper* can complement any of the missing recombination and repair genes in Glomeromycota. Experimental validation of *Glomhopper* activity may be challenged by the multinucleate state of Glomeromycota and known issues with genetic modification in these organisms [83]. Once methods are established, silencing of *Glomhopper* copies may provide valuable information on the role of DUF3504 in fungal biology and its evolutionary significance.

Importantly, YR-encoding elements have also been implicated in large-scale chromosomal rearrangements and horizontal transfer events, which may contribute to the adaptability of fungi in changing environments. Several studies suggest that *Starship*-like elements and other YR transposons can mobilise neighbouring genomic regions, carrying with them accessory genes linked to pathogenicity, secondary metabolism, or symbiosis [17, 80]. Whether DUF3504 are also involved in such rearrangements is not clear by now. There is however a clear proliferation of *Glomhopper* copies in Gigaspora genomes with hundreds spread over the assembly. The question on the impact of *Glomhopper* elements on the genomic architecture and possible rearrangements will be more feasible to answer when the current challenges with the fragmented assemblies of Glomeromycota high repetitive regions will be solved. The ubiquity of *Glomhopper*s in displaced contigs and repeat-rich genomic regions suggests a genomic location typical of most transposable elements in fungi, particularly those with a two-speed genome architecture.

## Conclusions

Fungi, and in particular Glomeromycota, have a unique family of YR-type of elements labelled as *Glomhopper*s. These elements have a single orf encoding a DUF3504 domain with a complete active site reassembling the *Cre* integrase of P1 bacteriophage. *Glomhoppers* are distantly related to *Cryptons* co-opted as ZMYM3 genes in animals. Sequence analyses indicate that *Glomhoppers* form several distinguishable subgroups or subfamilies, with most copies retaining the conserved catalytic residues, suggesting potential recombinase activity. In *Gigaspora* assemblies there are hundreds of *Glomhopper* copies, reflecting both bursts of transposon activity and lineage-specific expansions. Most *Glomhopper* are predicted to contain introns, which may reflect post-integration structural diversification and include both degenerating copies and candidates for incipient functional co-option. However, in the analysed transcriptomic datasets transcriptional activity is 2–8 times higher in intronless copies.

## Methods

### Discovery of *Glomhoppers*

The starting point was to collect all DUF3504 homologs. Using the METAEUK tool [35], we conducted an extensive analysis of 72 Glomeromycota genomes sourced from the NCBI database to identify regions similar to DUF3504. Our curated set of DUF3504 sequences served as the query for this analysis. The initial screening yielded approximately 74,000 regions that exhibited similarities to DUF3504.

To ensure the accuracy of our findings, we cross-checked the resulting 74,000 regions using HMMER, specifically employing the hmmsearch function against the DUF3504 HMM profile with an E-value cutoff 10e-2. This verification process refined our dataset, leaving us with 9,609 original hits deemed suitable for further analysis. These hits included both complete and partial DUF3504-containing sequences. Among these, a subset of approximately 1,800 elements was classified as *Glomhoppers* based on the presence of DUF3504 domains in a transposon-related genomic context and characteristic sequence features. Within this set, only a fraction retained a complete DUF3504/YR open reading frame, while the majority of copies were truncated or fragmented.

### Taxonomic distribution

The overall taxonomic distribution of proteins containing the DUF3504 domain was analysed using a range of bioinformatics tools: BLASTP, TBLASTN [7]. More specialised tools (HMMER, which uses hidden Markov models to identify homologous sequences, and PHMMER, a variant of HMMER designed to search for protein sequences) were used to detect the presence and distribution of DUF3504 domains in different species [11]. The TBLASTN tool from the Mycocosm portal was used to investigate the taxonomic distribution in Mucoromycota fungi (Nordberg et al. [52]).

### Detection of structural similarities to known tyrosine recombinases

Structural similarities were investigated using models available in the AlphaFold database specifically for fungal proteins, such as those of *Rhizophagus* [77]. In addition to the fungal models, representative species from other taxonomic lineages were also analysed, including *Xenopus* (a frog species), humans, *Lottia* (a marine mollusc) and *Mytilus* (a bivalve). To further explore these relationships, comparisons were made with structures from the Protein Data Bank (PDB) using the Dali server, a tool commonly used to identify structural similarities between proteins [22]. This approach provides a comprehensive picture of the behaviour and divergence of the DUF3504 domain in different species. Visualisation of protein structures was done in ChimeraX [45].

### Flanking repeats

A number of tools were used to identify transposon-flanking repeats (and thus determine their length): Generic Repeat Finder [68], RepeatMasker [69], LTR_retriever [59], LtrDetector [76], LTRpred [10], IRF [81], einverted from the EMBOSS server [65], Ugene [56], Starfish (with an in-house DUF3504 HMM built from fungal sequences) (Gluck-Thaler and [18] and minimap2 [36, 37].

### Sequence clustering and estimation of non-redundant Glomhopper loci

To assess sequence redundancy and approximate Glomhopper sequence diversity, nucleotide-level clustering was performed using CD-HIT-EST at 80%, 90%, and 95% identity thresholds. High-stringency clustering (95%) was run using:

$$\begin{aligned}cd&-hit-est-i\;input.fasta-o\;output.fasta\\&-c\;0.95-G\;1-g\;0-n\;8\end{aligned}$$

Where -c defines sequence identity, -G 1 enforces global alignment, -g 0 disables greedy incremental clustering, and -n 8 sets the word size for high-identity comparisons. Cluster outputs were used as a proxy for non-redundant sequence diversity across genomes. This approach does not resolve orthology between strains due to fragmented assemblies and variable genomic context of insertion sites; thus, highly similar sequences in different strains may represent orthologous insertions rather than independent events.

### Genomic neighborhood

The genomic neighborhood of *Glomhopper* elements were performed by mapping contig sequences from 72 Glomeromycota genomes on the CDD database [44] using rpstblastn [1] and METAEUK [35].

### Phylogenetic analysis

We added the recently identified *Starship* captain transposase [17] and the *Glomhopper* we identified to the phylogenetic analysis data set published by [64] [64]. In this way, a set of 198 YR-like sequences was obtained. The mentioned sequences were matched with the iterative local pairs algorithm implemented in Mafft [29]. The most suitable model for phylogenetic analysis was selected using ModelFinder implemented in IQ-TREE and according to the BIC criterion it was the VT + R5 model. Maximum likelihood analysis was carried out using IQ-TREE online version [73]. To assess the statistical reliability of the estimated phylogenies, branch support was calculated using bootstrap analysis and SH-aLRT branch test. The phylogenetic tree was graphically processed in iTol [34]. For phylogenetic reconstruction, a limited number of representative full-length *Glomhopper* sequences were selected to ensure reliable alignment and tree inference.

### DUF3504-*Glomhopper* clustering and classification

72 genomes from Glomeromycota were downloaded (19.04.2023) from NCBI and searched using RPS-tBLASTn for sequences matching the DUF3504 domain profile from the CDD database. This search initially retrieved 9,609 DUF3504-containing hits. The sequences were clustered with CD-HIT at 95% identity to reduce redundancy, resulting in 8,997 non-redundant sequences. For the CLANS analysis, 1,594 representative sequences were selected based on length (> 50 amino acids) and RPS-tBLASTn E-value (≤ 0.005). CLANS was then run with an E-value threshold of 1e-2 [12] to visualize the sequence relationships. This analysis provided a graphical summary of the diversity of proteins classified so far as DUF3504 in *Glomeromycota* and *Mucoromycota* fungi. Results included in the supplement. We enriched protein sequences corresponding to YR protein domains from the Ribeiro et al. article [64]—*DIRS* (*DIRS*-like, *PAT*-like, *Ngaro*-like, and *VIPER*-like), *VIPER*, *TATE* with other YR family proteins with reference DUF3435 sequences (*Starship*), *Cryptons* with RepBase mapping to DUF3504 and reference DUF3504 human and fungal sequences and grouped them together using CLANS.

### Expression and presence of introns

Using Metaeuk [35], we predicted exon–intron structures in a cluster of *Glomhopper* sequences. A total of 1,236 genomic DUF3504-containing loci were used for intron analysis in selected Glomeromycota, including both complete and truncated copies. Predicted introns were treated as candidate introns, and the number of introns ranged from 1 to 22, with the caveat that about 20% have no intron. Because Metaeuk infers gene models rather than directly identifying introns, all downstream analyses treated intron presence as a predicted feature rather than a definitive structural annotation. We did not show significant differences in the expression and methylation of variants having introns and those lacking them due to the few publicly available expression data in Mucoromycota. These loci include both *Glomhopper* elements and other DUF3504-containing sequences identified in the initial dataset.

*Glomhopper* copies were defined as DUF3504-containing sequences associated with transposon-like features, including genomic context and domain architecture. A subset of approximately 1,800 elements was classified as *Glomhoppers*, while the remaining sequences included partial, fragmented, or unclassified DUF3504 homologs. Orthologous insertions and recent transposition events were not explicitly distinguished; therefore, the reported copy numbers may include both shared orthologous elements and potentially recent insertions.

To support predicted exon–intron boundaries, spliced RNA-seq reads spanning exon–exon junctions were inspected, and only Glomhopper loci with transcriptomic support were retained for expression analyses. This step was included to reduce potential artefacts caused by fragmented or pseudogenized transposon copies that may lead gene prediction tools to introduce spurious introns.

In order to determine the expression levels within the two *Glomhopper* variants (classified based on predicted intron presence), we looked for publicly available transcriptomic experiments in Glomeromycota. We found data for six species; three of them with axenic culture transcriptome (*Funneliformis mosseae, Scutellospora calospora* and *Acaulospora morrowiae*) and three of them with condition-specific transcriptome (*Gigaspora rosea* DAOM 194757, *Gigaspora margarita* and *Rhizophagus irregularis* DAOM 197198) (Supplement S1, Table 7). The data was downloaded in the form of fastq files from the ENA server. They were quality checked using FASTQC (v0.11.8) [2] adapter-trimmed using fastp (v0.19.6) [8], aligned to the reference genomes (downloaded from NCBI Datasets) of the respective organisms using Hisat2 (v2.1.0) [30]. The alignment produced large-sized SAM files that were compressed into binary file (BAM) format using samtools (v1.10) [38]. The transcripts corresponding to the start and end coordinates of intron-containing and intronless variants were extracted from the BAM files and converted into BED format using an in-house script. StringTie (v2.1.3b) [61] was used to calculate the TPM (Transcript Per Million) values of the extracted transcripts. The average TPM was calculated for each dataset and values < 1 were regarded as statistically not significant and removed. The Wilcoxon rank sum test for intron and intronless transcripts was performed in R and violin plots were generated using violin function in RStudio.

## Supplementary Information

Supplementary Material 1.

## Data Availability

Materials from the supplement are available in the Zenodo repository: https://doi.org/10.5281/zenodo.19663248 All identifiers, *Glomhopper* loci and genomic assemblies are listed in **Supplementary file S1**: **Table 1.** List of genomes – list of Glomeromycota genomes used in the study **Table 2.** Raw data RPS-tblastn – raw data from RPS-tblastn analysis (against CDD profile—CDD:432,262) **Table 3.** Genome neighbourhood – genomic surroundings of the DUF3504 domain—*Glomhopper* – identified by RPS-tblastn **Table 4.** Starfish results – results from detecting repeats flanking a new transposon **Table 5.** METAEUK results – METAEUK results analyzed by HMMer against the DUF3504 profile **Table 6.** minimap2 results – alignment of full length copies on *Glomeromycota* genomes to the *Rhizophagus irregularis* GCA_026210795.1 (ASM2621079v1) reference genome **Table 7.** Transcriptomics data – *Glomhopper* expression levels in different *Mucoromycota* species under stress and non-stress conditions.
